# Influenza vaccination coverage in healthcare workers during the 2014-2015 season

**DOI:** 10.1186/2047-2994-4-S1-P18

**Published:** 2015-06-16

**Authors:** S Chyderiotis, E Seringe, K Blanckaert, L Guet, Z Kadi, P Astagneau

**Affiliations:** 1 North France healthcare Infection Control Centre (CClin Paris-Nord), PARIS, France

## Introduction

In France, influenza vaccination coverage remains poor among healthcare workers (HCWs) despite annual vaccination campaigns in healthcare institutions.

## Objectives

To evaluate the influenza vaccination coverage in HCWs in Northern France and to describe information and vaccination campaigns.

## Methods

A descriptive study was performed in healthcare institutions during February 2015. An online questionnaire was sent to the Infection Control Teams (ICT). They were asked to indicate their influenza vaccination coverage and the institutional means used to inform and vaccinate HCWs against influenza.

## Results

A total of 259 healthcare institutions (35%) answered. The overall influenza vaccination coverage for medical and non-medical staff reported during winter 2014-2015 was 19.7% [14.5-25.1] (n=219). The coverage was 36.3% (n=159) among doctors and midwives and 19.9% (n=169) among nurses and assistant nurses.

An information campaign was organized in 96% of healthcare institutions. Different communication mediums were used such as posters (76%), information on the hospital website (31%), individual e-mail (29%) or letter (23%), specific meetings (27%). The majority of healthcare institutions used 1 or 2 mediums (67%) whereas one third used 3 or more.

A vaccination campaign was organized in 98% of healthcare institutions. It was mostly led by the Occupational health Team (OHT) (39%) and/or the ICT (48%), but also by others (36%) such as the hospital pharmacy and the administration. The HCWs could be vaccinated directly by their colleagues (57%), the OHT (51%), a designated mobile team of HCWs (16%) and/or the ICT (9%). In 14% of cases, other options were implemented. The majority (62%) of healthcare institutions used one mean of vaccination, whereas 30% used 2 and only 8% used 3 or more. The night staff had access to vaccination means in 66% of healthcare institutions organizing campaigns.

## Conclusion

The influenza vaccination coverage is consistent with those previously reported in France and other countries. Despite most of the healthcare institutions organizing information and vaccination campaigns, it is far from the 75% recommended by the French health authority. Efforts should be made to enhance the vaccination coverage in order to prevent healthcare-associated influenza.

## Disclosure of interest

None declared.

